# Meloxicam versus Celecoxib for Postoperative Analgesia after Total Knee Arthroplasty: Safety, Efficacy and Cost

**DOI:** 10.5435/JAAOSGlobal-D-22-00032

**Published:** 2022-04-05

**Authors:** Amer Haffar, Yale A. Fillingham, Leigham Breckenridge, D'Andrew Gursay, Jess H. Lonner

**Affiliations:** From the Rothman Orthopaedic Institute, Thomas Jefferson University, Philadelphia, PA.

## Abstract

**Introduction:**

Nonsteroidal anti-inflammatory drugs (NSAIDs) are commonly used as part of multimodal analgesia in total knee arthroplasty (TKA). Selective cyclooxygenase (COX)-2 inhibitors (e.g., celecoxib) are believed to have fewer gastrointestinal (GI) adverse effects than nonselective NSAIDS. Meloxicam is less selective for COX-2 than celecoxib is and partially inhibits COX-1 at higher doses. Nonetheless, some surgeons prefer using nonselective NSAIDs because of their lower expense.

**Methods:**

Four thousand nine hundred ninety-four patients who underwent TKA between January 2015 and February 2020 and took either celecoxib (n = 3,174), meloxicam 15 mg/d (n = 1,819), or meloxicam 7.5 mg/d (n = 451) were studied. Mutlimodal postoperative analgesia protocols were otherwise similar. GI bleeding and wound complication incidence were determined, as well as average 30-day prescription costs.

**Results:**

GI bleeding incidence was similar in the three cohorts (*P* = 0.4). The incidence of wound complications did not significantly differ between the groups: 0.06%, 0.07%, and 0.22% in the celecoxib, meloxicam 15 mg/d, and meloxicam 7.5 mg/d groups, respectively (*P* = 0.06). Subsituting meloxicam for celecoxib results in an average savings of $183 per prescription.

**Discussion:**

Meloxicam used at higher doses (15 mg/d) does not markedly increase the risk of GI or wound complications associated with COX-1 inhibition and is less costly for multimodal analgesia after TKA.

Total knee arthroplasty (TKA) for the treatment of degenerative joint disease provides patients with an improved quality of life and better functional status; however, the procedure can result in moderate-to-severe postoperative pain.^1^ Historically, opioid medications have been the cornerstone of postoperative analgesia after TKA, but our improved understanding of pain physiology and transmission helped to usher in the era of multimodal analgesia.^2^ The premise of multimodal analgesia is to use nonnarcotic medications such as acetaminophen, gabapentinoids, and nonsteroidal anti-inflammatory drugs (NSAIDs), among others, to reduce opioid-related adverse effects while providing improved pain management.^3^ For example, the tissue damage during TKA causes the release of arachidonic acid, the precursor to prostaglandins, resulting in a hyperalgesiac response from the peripheral nerves.^1,4,5^ Because NSAIDs block the conversion of arachidonic acid to prostaglandins, causing a subsequent reduction in the hyperalgesiac response, NSAIDs can be used to reduce postoperative pain as an alternative to opioid medications.^1,4,5^

Nonsteroidal anti-inflammatory drugs are categorized based on their ability to selectively inhibit the cyclooxygenase (COX)-2 isoform that is responsible for the production of prostiglandins. Nonselective NSAIDs (eg, ibuprofen) provide relatively equivalent inhibition of the COX-1 and COX-2 isoforms while selective COX-2 NSAIDs are subcategorized as highly selective (eg, celecoxib) and preferentially selective (eg, meloxicam) COX-2 NSAIDs.^6^ Although meloxicam does have a preference for binding with COX-2, it also inhibits COX-1 at the higher dosage regimens of 15 mg daily.^7-9^ Because COX-1 is constitutively expressed throughout the body and plays a role in platelet function, protection of gastric mucosa, and renal function, it resulted in the development of the selective and preferentially selective COX-2 NSAIDs with the intention to limit the negative effect of COX-1 inhibition.^5,9^ Nevertheless, realizing the full benefit of highly selective COX-2 inhibition while avoiding the complications associated with COX-1 inhibition has not come to fruition.^5^

The purpose of this study was to compare the complication rates related to COX-1 inhibition between those who receive either a nonselective (meloxicam 15 mg/d), preferential COX-2 (meloxicam 7.5 mg/d) or a highly selective COX-2 (celecoxib) NSAID after primary TKA and determine whether meloxicam, at a dose of 15 mg/d, is more cost-effective when considering the potential cost of increased complications related to COX-1 inhibition.

## Materials and Methods

After receiving exemption from our institutional review board, we searched our institutional database to identify patients who received a primary TKA between January 2015 and February 2020 who used celecoxib or meloxicam for postoperative analgesia. Because meloxicam acts as a preferential COX-2 inhibitor at lower doses and a nonselective NSAID at higher does, patients taking meloxicam were further stratified into a 15 mg/d meloxicam cohort and 7.5 mg/d meloxicam cohort, and as such, three cohorts of patients were analyzed.^10^ A total of 4,994 patients were included in this study who took either celecoxib (n = 3,174), meloxicam 15 mg/d (n = 1,369), or meloxicam 7.5 mg/d (n = 451) for postoperative TKA analgesia. Baseline characteristics between the three groups were similar with the exception of a slightly higher percentage of men in the celecoxib cohort, slightly higher BMI in the meloxicam cohorts, and lower CCI in the meloxicam 7.5 mg/d cohort (Table 1); however, the age-adjusted CCI demonstrated similarity among the three cohorts. Patients were either prescribed meloxicam 7.5 mg/d, meloxicam 15 mg/d, or celecoxib because of surgeon preference. Surgeons tended to choose one medication and one dosage regimen and did not switch over to another medication based on perceived risk. Patient pain protocols were otherwise similar among the three groups, and all patients were prescribed with a proton-pump inhibitor as a part of the standard of care at our institution. Patients who did not fill their prescriptions or were found to not be taking the prescription were excluded. After reviewing discharge records and patient charts, a total of 4,994 patients were included in the study.

**Table 1:** Summary of Patient Demographics

Factor	Celecoxib	Meloxicam 15 mg/d	Meloxicam 7.5 mg/d	*P*
	N = 3,174	N = 1,369	N = 451	
Age	65.0 (±9.01)	64.5 (±8.81)	65.1 (±8.73)	0.2
Sex				**0.01**
Female	58.9%	63.3%	63.6%	
Male	41.1%	36.7%	36.4%	
BMI	30.8 (±5.17)	31.1 (±5.35)	31.3 (±5.05)	**0.04**
CCI	0.69 (±1.03)	0.74 (±1.12)	0.58 (±0.95)	**0.03**
Age adjusted CCI	2.74 (±1.43)	2.77 (±1.46)	2.65 (±1.35)	0.4
History of chronic renal disease (CRD)				0.8
Yes	0.62%	0.79%	0.49%	
No	99.4%	99.2%	99.5%	
History of peptic ulcer disease (PUD)				1.0
Yes	0.2%	0.1%	0%	
No	99.8%	99.9%	100%	
VTE prophylaxis drug				**<0.001**
Aspirin	86.9%	86.0%	95.9%	
Other	13.1%	14.0%	4.1%	

VTE = thromboembolic event, BMI = Body Mass Index, CCI = Charlson Comorbidity Index

Values are reported in means and SD, with the exception of sex and VTE Prophylaxis. Bold *P* Values <0.05 are deemed to be statistically significant.

The primary outcomes of our study were the incidence of hematoma, generalized bleeding, gastrointestinal (GI) bleeding, generalized wound complications, acute kidney injury, and manupulations under anesthesia. Generalized wound complications were defined to include incidents of wound dehiscence (excluding those from falls), wound drainage, incisional bleeding, and delayed wound healing. Acute kidney injury was defined as an abrupt decrease in glomerular filtration rate and increase in creatinine and BUN. Our institutional nurse navigator program that tracks postoperative complications was queried to identify patients who developed any 90-day complications. The implementation of our institutional nurse navigator program has proven effective in detecting postoperative complications and even reducing the episode of care costs after joint arthroplasty.^11^ Venous thromboembolic event (VTE) prophylaxis chemical agent use was also noted to control for any potential source of confounding for postoperative complications. Patients were recorded as taking either aspirin or nonaspirin (rivaraxoban, coumadin, enoxaparin, apixaban, clopidegerol, or dabigatran). Aspirin, the agent that is most commonly used at our institution for VTE prophylaxis, is an irreversible COX-1 inhibitor,^12^ hindering platelet synthesis and potentially compromising gastorintestinal and renal health. Patients were deemed to be nonaspirin users if they were found to be taking either rivaraxoban, coumadin, enoxaparin, apixaban, clopidegerol, or dabigatran and were analyzed together. Medications such as factor Xa and direct thrombin inhibitors may be associated with increased bleeding and wound complication rates^13^ while aspirin may limit wound complications and bleeding.^14,15^ Average retail prices for a 30-day supply of 15 mg of meloxicam ($32.99) and 200 mg of celecoxib ($215.99), as of February 2021, were obtained using www.WebMD.com/rx/drug-prices. Informal cost analysis was done by comparing the average retail price of a 30-day supply of 15 mg of meloxicam and 200 mg of celecoxib, the two most commonly prescribed dosages for those medications at our institution, to calculate the savings associated with substituting meloxicam 15 mg/d for celecoxib 200 mg/d in postoperative TJA pain protocols. Continuous data are presented as mean (SD), and categorical data are presented as cell count (percent of total count). T-tests or analysis of variance tests were used to calculate *P* values for continuous data, and chi-square testing was used for categorical data. *P* values less than 0.05 was used to determine significance. All statistical analyses were done using R Studio (Version 3.6.3, Vienna, Austria).

## Results

Patients in the three cohorts had similar incidences of renal and peptic ulcer disease and were similar for GI and renal risk (Table 1). Aspirin BID usage for VTE prophylaxis was 96% in the meloxicam 7.5 mg/d cohort, 86% in the meloxicam 15 mg/d cohort, and 87% in the celecoxib cohort (*P* < 0.001). No statistical difference in complication rates were found between the three cohorts (Table 2).

**Table 2:** Summary of the Complication Data for Our Study

Factor	Celecoxib	Meloxicam 15 mg/d	Meloxicam 7.5 mg/d	*P*
Hematoma	6 (0.19%)	1 (0.07%)	0 (0.00%)	0.8
Generalized bleeding	2 (0.06%)	1 (0.07%)	0 (0.00%)	1.0
GI bleeding/ulceration	2 (0.06%)	1 (0.07%)	1 (0.22%)	0.4
Generalized wound complications	18 (0.57%)	5 (0.37%)	3 (0.67%)	0.6
Acute kidney injury	0 (0%)	0 (0%)	0 (0%)	1.0
Manupulations under anesthesia	190 (5.99%)	76 (5.55%)	22 (4.88%)	0.6

## Discussion

A large impediment to the wider adoption of oral NSAIDs for postoperative primary TKA analgesia are concerns regarding the GI and renal adverse effects associated with COX-1 inhibition from nonselective NSAIDs, especially with the increased utilization of postoperative IV ketorolac, IV corticosteroids, and VTE prophylaxis of aspirin.^9^ Currently, limited evidence is available regarding the safety profile of nonselective NSAIDs after discharge from primary TJA. In light of the opioid epidemic, a working committee of the American Association of Hip and Knee Surgeons, American Academy of Orthopaedic Surgeons Hip Society, Knee Society, and the American Society of Regional Anesthesia and Pain Medicine have suggested that the safety of nonselective NSAIDs after primary TKA be investigated.^16^

Therefore, we sought to investigate for any potential differences in COX-1 inhibition-related postoperative complications between nonselective NSAID dosing of meloxicam, preferential COX-2 NSAID dosing of meloxicam, or highly selective COX-2 NSAID dosing of celecoxib. Our findings suggest that using meloxicam at either a nonselective (15 mg/d) or preferential COX-2 (7.5 mg/d) dose as a part of a postoperative multimodal TKA pain protocol does not seem to result in an increased incidence of postoperative GI bleeding compared with the usage of celecoxib, *P* = 0.4, considering that patients in the three cohorts had similar GI risk profiles: 0.2%, 0.1%, and 0% of patients in the celecoxib, meloxicam 15 mg/d, and meloxicam 7.5 mg/d cohorts had a history of peptic ulcer disease (*P* = 1.0). These findings did not agree with our hypothesis because we expected patients on meloxicam, particularly when consuming 15 mg/d, to have a greater incidence of GI bleeding and ulceration based on meloxicam being a nonselective NSAID at higher doses.

Currently, there is a paucity of data comparing the incidence of GI complications between selective COX-2, preferential COX-2, and nonselective NSAIDs with inconsistency in the reported literature. A study of the general population in England showed that the usage of meloxicam compared with celecoxib resulted in an increased incidence of GI complications.^17^ After adjustment for age, sex, medical history of upper GI complications, and NSAID usage within 3 months of the study period, celecoxib was found to result in a relative rate reduction in symptomatic GI events and upper GI complications.^17^ In the realm of joint arthroplasty analgesia, a high-quality clinical trial showed that a nonselective NSAID (lornoxicam) resulted in a higher incidence of symptomatic GI complications than a selective COX-2 NSAID,^18^ but did not reach statistical significance. Although we only reported clinical GI complications, and not symptomatic GI complications, there seems to be an agreement with the findings of our study. The results of our study also agreed with that of Rawal et al^19^ because their results suggested that there was no increased incidence of adverse GI events between a selective COX-2 NSAID, etoricoxib, and nonselective NSAID, ibuprofen. Nevertheless, because of the rarity of upper GI complications associated with NSAIDS,^16^ clinical trials may not report this extensively and may be underpowered.

The incidence of hematoma and generalized bleeding was found to be equivalent among patients in the three cohorts, *P* = 0.8, and *P* = 1.0, respectively. Limited data are currently available to suggest whether nonselective NSAIDS are inferior to selective COX-2 NSAIDs for postoperative bleeding after primary TJA. Nevertheless, our findings agree with those of another clinical trial,^19^ which showed that there was no difference among patients in the treatment groups comparing etoricoxib, ibuprofen, and placebo for the incidence of postoperative bleeding events after TKA. Patients in that study received the intervention for 7 days postoperatively and were not on any medication for VTE prophylaxis. Their results were underpowered to determine bleeding risk and should be interpreted with caution.

We report no incidents of acute kidney injury among our patients (*P* = 1.0). These results are promising because we had a relatively large patient cohort compared with previous clinical trials and that patients in the three cohorts were similar for renal risk (*P* = 0.8), we support conducting additional studies to determine whether nonselective NSAIDs result in an increased incidence of acute kidney injury. There may be a possibility that a larger patient population may be needed to determine any differences for this rare yet clinically devastating event. Currently, there are no high and moderate quality clinical trials that compare the incidence of acute kidney injury between selective COX-2 NSAIDs and nonselective NSAIDS in the realm of joint arthroplasty.^9^ In one systematic review and meta-analysis, meloxicam was found to result in an increased risk of acute renal failure compared with nonusers; however, these results did not reach statistical significance and this may be attributed to small sample size.^20^ Celecoxib was found to impart an increased risk of acute renal failure compared with nonusers; however, this did not approach statistical significance as well.^20^ Nevertheless, just like our study, Ungprasert et al^20^ did not find any difference in the overall risk for developing acute renal injury.

With our findings suggesting no difference in the incidence of postoperative complications among patients using low-dose meloxicam (7.5 mg/d), high-dose meloxicam (15 mg/d), and celecoxib, formal cost-analysis was not done because it is evident that meloxicam usage would demonstrate cost-savings. Using the values of the average retail medication prices in 2020, we found the cost for a 30-day supply of 15 mg of meloxicam to be $32.99, whereas the cost of a 30-day supply of 200 mg of celecoxib to be $215.99. If patients were to use 15 mg meloxicam daily for postoperative TKA analgesia rather than 200 mg celecoxib daily, this would result in a saving of $183 per prescription on average. Based on the finding that 9% of the patients were prescribed celecoxib for postoperative pain control at our institution, if this figure were to be applied to the TKA population in the United States, approximately 700,000, prescribing meloxicam in place of celecoxib would generate a savings of $12 million to the healthcare system.

Our study contains all the weaknesses that are present in retrospective reviews. Because we established cohorts based on what TKA patients were prescribed, the retrospective nature of the study means that patients who were prescribed may not have been taking celecoxib or meloxicam as instructed. Nonetheless, we reviewed the discharge medications of those who had a discharge record present in our system and whether celecoxib or meloxicam use was mentioned in patient chart note search to improve the accuracy of our data. About our primary end point, GI bleeding/ulceration, we were underpowered because of the low incidence of these complications in our cohort. The low incidence of GI complications and thus the underpowered findings may be attributable to the rarity of the complication.^16^ At our institution, the historical rate of GI complications after TJA from 2008 to 2019 was 0.6% (102/17,402 patients), and peptic ulcer disease was found to be a notable risk factor.^21^ In our study, 0.2%, 0.1%, and 0% of patients in the celecoxib, meloxicam 15 mg/d, and meloxicam 7.5 mg/d cohorts, respectively, had a prior history of peptic ulcer disease (*P* = 1.0), suggesting that patients in our study had low GI risk in general. Furthermore, our institutional guidelines limit the prescription of NSAIDS to patients with serious GI risk. Therefore, we believe that these variables, taken together, should not detract from the findings of our study and that these results offer the best available evidence on NSAIDS and postoperative complications in TKA. We would encourage prospective studies to corroborate our findings. We also could not evaluate the analgesic effectiveness of the medications used at various dosages. Nevertheless, clinical practice guidelines dictate that irrespective of COX-1/COX-2 selectivity, there is no difference in analgesic effectiveness among NSAIDs.^18,19,22^ Although we used average retail costs, in practice, the cost of prescriptions may vary depending on insurance coverage, rebate, and discount programs at pharmacies. The strengths of our study include having the largest retrospective comparative study of the safety profile of NSAIDS in the realm of TJA and having a large cohort size of patients, showing that our results have external validity.

## Conclusion

Although the importance of a patient's medical history has been stressed when prescribing NSAIDS, particularly higher doses of meloxicam, it has an equivalent safety profile to celecoxib after primary TKA. Meloxicam usage, particularly at higher doses does not seem to increase the risk of complications potentially related to COX-1 inhibition. Therefore, meloxicam may be considered a safe and low cost alternative to celecoxib for TJA postoperative analgesia.
